# Cobalt sulfide flower-like derived from metal organic frameworks on nickel foam as an electrode for fabrication of asymmetric supercapacitors

**DOI:** 10.1038/s41598-024-56689-9

**Published:** 2024-03-13

**Authors:** Farzaneh Nasiri, Lida Fotouhi, Saeed Shahrokhian, Mohammad Zirak

**Affiliations:** 1 Department of Analytical Chemistry, Faculty of Chemistry, Alzahra University, Tehran, Iran; 2 Analytical and Bioanalytical Research Centre (ABRC), Alzahra University, Tehran, Iran; 3 Department of Chemistry, Sharif University of Technology, 11155–9516, Tehran, Iran; 4 Department of Physics, Hakim Sabzevari University, P. O. Box 961797647, Sabzevar, Iran

**Keywords:** Hybrid supercapacitor, Metal–organic frameworks, Cobalt sulfide nanosheet, Energy storage, Supercapacitors, Analytical chemistry

## Abstract

Metal–organic frameworks, as a kind of advanced nanoporous materials with metal centers and organic linkers, have been applied as promising electrode materials in energy storage devices. In this study, we are successfully prepared cobalt sulfide nanosheets (CoS) derived from the metal–organic framework on nickel foam (NF). The prepared electrodes are characterized by scanning electron microscopy, transmission electron microscopy, X-ray diffraction, X-ray photoelectron spectroscopy, energy-dispersive X-ray spectroscopy, Brunauer–Emmett–Teller and Barrett-Joyner-Halenda and electrochemical methods like voltammetry, galvanostatic charge–discharge curve and electrochemical impedance spectroscopy**.** The CoS/NF electrode demonstrates a high specific capacity of 377.5 mA h g^−1^ (1359 C g^−1^) at the current density of 2 A g^−1^, considerable rate performance and excellent durability (89.4% after 4000 cycles). A hybrid supercapacitor is assembled using CoS/NF as the positive electrode and activated carbon as the negative electrode, it shows a high energy density of 57.4 W h kg^−1^ at a power density of 405.2 W kg^−1^. The electrochemical results suggest that the CoS nanosheet arrays would possess excellent potential for applications in energy storage devices.

## Introduction

The depletion of fossil fuel reserves and the environmental consequences of greenhouse gas emissions have prompted a growing global need for the development of sustainable energy sources worldwide^1,2^. Supercapacitors are a class of energy storage devices which operate by rapid electrostatic or Faradaic electrochemical mechanisms. They are composed of positive and negative electrodes immersed in an electrolyte and divided by an ion permeable, electronically insulating separator. Although the general charge storage and performance principles of supercapacitors resemble those of conventional capacitors, their specific capacitance and energy density are enhanced by a factor of 100,000 or more compared to regular capacitors^1^. Numerous energy storage devices have been developed for application in energy storage. However, supercapacitors, also referred to as ultracapacitors, have gained significant attention due to their high power densities, extended life cycle, rapid charge–discharge rates, substantial capacitance, and reliable operational safety. Nevertheless, the limited energy density of supercapacitors presents a significant obstacle to their practical implementation^3–6^. One of the greatest challenges in energy storage is the design of energy storage systems from tolerable sources with high energy capacity and power capability. Hence, it is important to search for other advanced electrode materials with high capacity, low cost and eco-friendly^4,7,8^. There has been a significant increase in research efforts to develop advanced electrode active materials due to the growing demand for high performance, affordable, and safe energy storage devices^6^. The performance of supercapacitors can be enhanced by the electrical conductivity of both the electrode materials and the substrate. Furthermore, the operating potential window of a supercapacitor can be expanded by using ionic liquid electrolytes or fabrication of asymmetric supercapacitors with different positive and negative electrode materials^4,7^.

Metal hydroxides, oxides, and sulfides have been used as redox-type electrode materials with high capacitance and novel catalysts with low cost and great catalytic activity. In comparison to similar metal-based compounds, metal sulfides show higher electrical conductivity, more desirable mechanical properties and more appropriate thermal stability^2,9,10^.

Metal–organic frameworks (MOFs) are a new class of nanoporous compounds consisting of metal ions and organic linkers^11,12^. Compared with traditional porous materials, MOFs have advantages including various scaffold structures, adjustable pore sizes, large specific surface areas, and abundance of active sites. Interest in this new type of porous materials is growing rapidly as their potential applications in many fields are introduced. MOFs and their derivatives have also been applied in the field of electrochemical energy storage. Therefore, MOFs and their derivatives with good stability and high conductivity are highly preferred in the development of supercapacitor electrodes^13,14^.

In comparison with conventional metal sulfides, MOF-derived metal sulfides could largely inherit the characteristics such as large surface area, tailored porosity and composition diversity of the original MOF materials, which have been widely applied in energy storage applications^2,15^. Recently, metal sulfides synthesized by MOF templates with high conductivity and various structures have attracted major attention. For instance, Li et al. reported the fabrication of MOF-derived Cu_7_S_4_/C nanoparticles, which showed a capacitance of 1323.6 F g^−1^ at 1 A g^−1^, and a capacitance retention of 88.8% after 5000 cycles^16^. Furthermore, Nio et al. fabricated MOF-derived copper sulfide polyhedrons on a carbon nanotube structure, which showed a specific capacitance of 606.7 F g^−1^. In addition, an asymmetric supercapacitor based on HKUST-1 derived CuS polyhedrons and nitrogen-doped carbon polyhedrons into carbon nanotube as the positive and negative electrodes, respectively, provided an energy density of 38.4 W h kg^−1^ at a power density of 750 W kg^−1^^17^. Li et al. reported the synthesis of NiS_2_/ZnS nanospheres with a specific capacitance of 1198 F g^−1^ at a current density of 1 A g^−1^. When the synthesized electrode was used as the positive electrode for supercapacitor, it demonstrated an energy density of 28 W h kg^−1^ at a power density of 20 kW kg^−1^^18^. In another work, Chamesh et al. prepared ZIF-derived binary ZnS/CoS. The prepared electrode showed a specific capacitance of 1646 F g^−1^ at a current density of 1 A g^−1^^19^. Qu et al. reported a specific capacitance of 1571.8 F g^−1^ at a current density of 1 A g^−1^ for r-Ni_3_S_2_ on NF^20^. In the earlier work, Jia et al. synthesized CoS_2_ hollow dodecahedrons with different morphologies. They reported that the flower-like Co-S delivered a specific capacitance of 377.5 mA h g^−1^ at 1 A g^−1^, which was higher than the corresponding value for CoS_2_ hollow dodecahedrons (375.2 C g^−1^)^21^.

NF has been widely utilized as a substrate and current collector in Ni based electrodes due to its favorable characteristics, such as cost-effectiveness and high conductivity, which surpass those of other metallic substrates. Hence, there is a significant need to develop electrodes, which do not require a binder, allowing for direct connection of active materials to the conductive NF. The application of MOF derived compounds has been shown to enhance electrical conductivity and offer a substantial electrode surface area, thereby promoting efficient faradic reactions. These finding have sparked significant interest in the development of techniques for the preparation carbonaceous compounds with precisely controlled size and morphology^2,11,14^.

The fixation of CoS on a conductive substrate has been reported in various energy related applications (Table 1). Despite the reported success in the performance of the SC electrodes, further investigation and optimization of the morphology of NF with CoS are necessary to enhance the performance of supercapacitors. The optimization of CoS through optimizing their morphology and some of their other properties via a straightforward, easily tunable, cost effective method is highly demanded. Significant enhancement in the capacitive and catalytic performances of CoS compounds can be achieved through the control of their morphologies and dimensions to a scale of a few nanometers^20–22^.

**Table 1:** Comparison of the electrochemical performance of the presented electrode with those of previously reported composites.

Electrode material	Precursor	3 electrode system		2 electrode system		Ref
		Capacity or capacitance	Cycle stability	Energy density	Power density	
Co_9_S_8_@C nanoparticles	Co-BTC	1672 F g^−1^ at 1 A g^−1^	100% retention after 4000	58 W h kg^−1^	1000 W kg^−1^	^23^
Co_9_S_8_ nanowire arrays	ZIF-67	4.44 F cm^−2^ at 1 A g^−1^	1.6 F cm^−2^ after 10,000	–	–	^24^
Co_2_S hollow dodecahedrons	ZIF-67	375.2 C g^−1^ at 1 A g^−1^	92.1% after 4000	52.1 W h kg^−1^	401 W kg^−1^	^21^
Fungus-like CoS	–	350 F g^−1^ at 1 A g^−1^		45.2 W h kg^−1^	1500 W kg^−1^	^25^
CoS	–	127 C g^−1^ at 1 A g^−1^		38 W h kg^−1^	533 W kg^−1^	^26^
Mxene/CoS_2_	–	1320 F g^−1^ at 1 A g^−1^	78.4% after 3000	28.8 W h kg^−1^	800 W kg^−1^	^27^
Co_9_S_8_	–	1056 F g^−1^ at 5 mV s^−1^		31.4 W h kg^−1^	200 W kg^−1^	^28^
Co–Ni-S	–	430.1 C g^−1^ at 1 A g^−1^	82% after 10,000	41.98 W h Kg^−1^	800.04 W kg^−1^	^29^
CoS	–	570 F g^−1^ at 1 A g^−1^	97.9% after 2000	15.58 W h kg^−1^	700.12 W kg^−1^	^30^
Flower-like CoS	Co-MOF	377.5 mA h g^−1^ (1359 C g^−1^) at 2 A g^−1^	89.4% after 4000	57.4 W h kg^–1^	405.2 W kg^−1^	This work

Herein, an asymmetric supercapacitor (ASC) has been assembled using CoS/NF and activated carbon (AC) as the positive and negative electrode, respectively. Briefly, Co-MOF nanosheets were first generated by the solvothermal method, followed by partial transfer to Co-S by the water bath process. Prior to the fabrication of NF/CoS//AC/NF ASCs, the electrochemical behavior of both positive and negative electrodes was evaluated via cyclic voltammetry (CV), galvanostatic charge–discharge (GCD) and electrochemical impedance spectroscopy (EIS). The electrochemical performance of the prepared electrode was investigated in a 3 M KOH electrolyte. The CoS electrode material showed a high specific capacity of 377.5 mA h g^−1^ (1359 C g^−1^) at the current density of 2 A g^−1^. The particular structure exhibited a prominent cycling stability of 89.4% after 4000 cycles. Moreover, the ASC exhibited a high energy density of 57.4 W h kg^−1^ at a power density of 405.2 W kg^−1^ with an excellent cycling stability (91.7% capacitance retention after 4000 cycles).

## Experimental

### Materials

All the reagents were of analytical grade and used without further purification. Cobalt nitrate hexahydrate (Co(NO_3_)_2_.6H_2_O), Na_2_S.9H_2_O, terephthalic acid (TPA), potassium hydroxide (KOH), ethanol, dimethylformamide (DMF), N-methyl-2-pyrrolidone (NMP), active carbon (AC), polyvinylidene difluoride (PVDF), and carbon black were obtained from Merck (Darmstadt, Germany, www.merck.com).

### Preparation of Co-MOF

Firstly, 0.244 g Co(NO_3_)_2_.6H_2_O and 0.170 g TPA were added to 35 mL DMF. Then the above solution was slowly added to 2.5 mL ethanol and 2.5 mL water, respectively. After stirring for a few minutes, the solution was transferred to a 50 mL Teflon lined autoclave, and then a piece of cleaned NF substrate was immersed into the reaction solution and heated up to 125 °C for 12 h. Eventually, the NF was washed several times with ethanol and water in turn and dried at 70 °C.

### Preparation of MOF-derived CoS

In a typical procedure, 0.3 g Na_2_S.9H_2_O was added to 35 mL deionized water and stirred for 10 min, and the solution was heated to 80 °C and held for 10 min. A piece of Co-MOF/NF arrays was inserted into the solution and kept for 10 min. Finally, the as-obtained NF was washed using ethanol and water in turn to remove the impurities and dried at 80 °C. The final product was the CoS electrode. The mass loading of CoS electrode is about 2 mg cm^−2^.

### Electrochemical measurements

All electrochemical measurements were performed using a μ-Autolab TYPE Ш potentiostat/galvanostatic controlled via Nova, 2.1.2 software (https://cdn.discordapp.com/attachments/1202910306058108941/1202911765105606726/setup_v3.rar?uel=nova_2.1_download.zip). A three-electrode system with a NF substrate incorporating the active materials (Co-MOF and CoS), a saturated Ag/AgCl electrode, and a platinum plate were used as working, reference, and counter electrodes, respectively, in 3 M KOH as the supporting electrolyte. The electrochemical features were investigated via CV at different scan rates ranging 10 to 60 mVs^−1^, GCD with different current densities from 2 to 20 A g^−1^, and EIS in a frequency ranging from 0.1 Hz to 100 kHz at the open circuit potential condition.

The specific capacity (mA h g^−1^) of the battery-type CoS/NF was calculated based on the Eq. (1):

1 $$\mathrm{Q }=\frac{{\varvec{I}}\boldsymbol{ }\int {\varvec{V}}{\varvec{d}}{\varvec{t}}}{{\varvec{m}}\boldsymbol{ }\times 3.6\boldsymbol{ }\times {\varvec{V}}}$$

where I (A) is current ∫V dt (s) is the area under discharge curve, m (g) is the mass of the electrodes, and V (V) is the potential window^22^.

The specific capacity (C g^−1^) of the battery-type CoS/NF was calculated based on the Eq. (2):

2 $${\text{C}}=\frac{{\varvec{I}}\int {\varvec{V}}{\varvec{d}}{\varvec{t}}}{{\varvec{m}}{\varvec{V}}},$$

where I (A) is current, ∫V dt (s) is the integrated area under discharge curve, m (g) is the mass of the electrodes, and V (V) is the potential window^31^.

### Fabrication of the ASC device

To assemble an ASC, as-obtained CoS/NF was utilized as the positive electrode. A mixture of AC, carbon black and PVDF with a mass ratio of 70:20:10 in NMP was pressed on the surface of a piece of NF as the negative electrode. The charge balance between CoS/NF and AC/NF electrodes assembling ASC devices was calculated according to Eq. (3).

3 $$\frac{{{\varvec{m}}}^{+}}{{{\varvec{m}}}^{-}}=\frac{{C}^{-}\boldsymbol{ }\times \boldsymbol{ }{\Delta {\varvec{V}}}^{-}}{{C}^{+}\boldsymbol{ }\times \boldsymbol{ }{\Delta {\varvec{V}}}^{+}},$$

where ∆V^+^ and ∆V^−^ are potential windows of positive and negative electrodes, C^−^ and C^+^ were estimated from CV curves of individual negative and positive electrodes^31,32^. The total mass loading of an asymmetric supercapacitor is about 3.8 mg cm^−2^.

The specific capacitance (F g^−1^) of CoS/NF//AC/NF electrodes ASC device was estimated from GCD measurements based on Eq. (4)**.**

4 $${C}_{a}=\frac{{\varvec{I}}\boldsymbol{ }\times \Delta {\varvec{t}}}{{\varvec{M}}\boldsymbol{ }\times \Delta {\varvec{V}}},$$

where I (A) is current, ∆t (s) is discharge time, M (g) is the total mass of the positive and negative electrodes (M = m_anode_ + m_cathode_), ∆V (V) is the potential window^33,34^.

The energy density (E, W h kg^−1^) and power density (P, W kg^−1^) of ASCs were calculated according to the below equations^33,35^:

5 $$\mathrm{E }=\frac{\mathbf{I}\int \mathbf{V}\mathbf{d}\mathbf{t}}{{\varvec{m}}}$$

6 $$\mathrm{P }=\frac{3600{\varvec{E}}}{\Delta {\varvec{t}}}.$$

The coulombic efficiency (η) of the electrode was calculated using the following equation^35^.

7 $$\upeta =\frac{{{\varvec{t}}}_{{\varvec{d}}}}{{{\varvec{t}}}_{{\varvec{c}}}},$$

where t_c_ and t_d_ are the charging and discharging times of the GCD curve, respectively.

### Instrumentation

Scanning electron microscopy (SEM, TESCAN MIRA3, Australia) and transmission electron microscopy (TEM) were utilized to analyze the morphology and structure of the prepared materials. The crystallinity and nanostructure of all samples were characterized using X-ray diffraction (XRD, X^'^Pert PRO MPD, PANalytical diffractometer, Netherlands using Cu Kα radiation). X-ray photoelectron spectroscopy (XPS, UHV analysis system) was used to determine the chemical state and the surface-bound functionalities utilizing Al K_α_ X-ray origin. Surface area and pore size distribution measurements were taken using Brunauer–Emmett–Teller (BET) and Barrett-Joyner-Halenda (BJH) methods, respectively**.**

## Results and discussion

### Structural and morphology characterization

**Figure 1:**
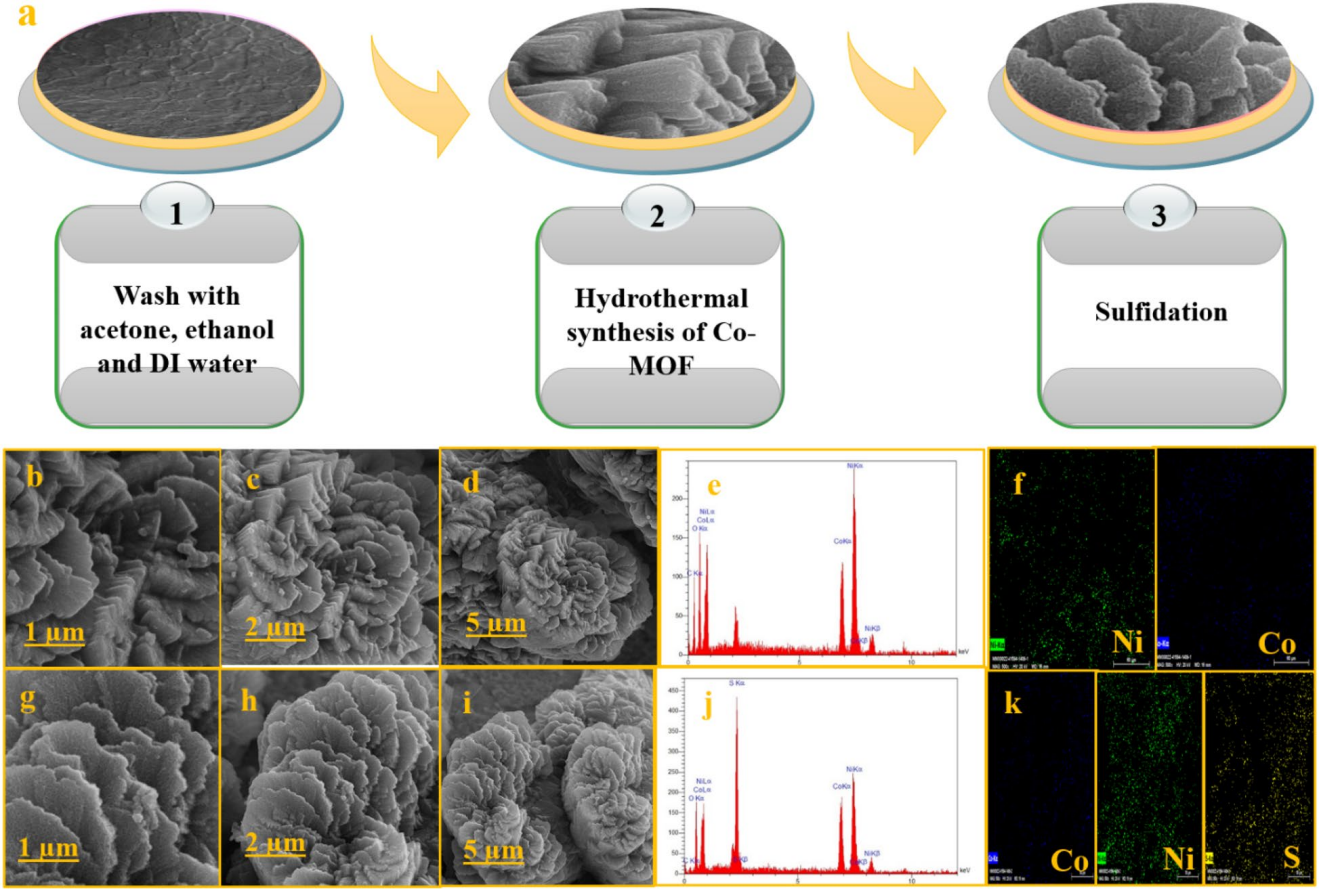
(**a**) Schematic representation of the fabrication of CoS, (**b**–**d**) SEM images of Co-MOF and (**g**–**i**) SEM images of CoS at scales of 1, 2 and 5 μm, (**e**,**j**) EDS pattern spectra Co-MOF and CoS, (**f**,**k**) the elemental mappings of Co-MOF and Co-S.

The stepwise synthesis of CoS nanosheet arrays on NF is depicted in Fig. 1a. The Co-MOF nanosheet arrays are first generated by the solvothermal method and then transferred to CoS by the water bath process at 80 °C, for 10 min. The Co^2+^ ions in Co(NO_3_)_2_.6H_2_O react with C_6_H_4_(CO_2_H)_2_ (TPA) in the solution to generate Co-MOF on the electrode. Afterward, during the sulfidation process in Na_2_S.9H_2_O solution, S^2−^ anion reacts with Co^2+^ in the Co-MOF to generate CoS (Co^2+^ + Na_2_S.9H_2_O → CoS + NaOH + H_2_O). The surface morphology of Co-MOF nanosheets and CoS was characterized by SEM and the results have been shown in Figs. 1b–d and g–i. The SEM images of Co-MOF nanosheets exhibit a flower-like morphology with smooth surfaces. After the sulfurization in Na_2_S solution, the color of Co-MOF changes from purple to black. The uniform distribution of CoS nanosheets on the surface of nickel foam confirms the retention of the nanoarray structure after sulfidation as shown in Fig. 1g. Furthermore, the nanosheets with crimp edges and wrinkled surfaces have become thinner and semi-transparent, and also their surface porosity has increased.

The EDS analysis of Co-MOF and Co-S shows that the prepared electrodes are composed of Co, Ni, and S elements (Figs. 1e,j), confirming the successful preparation of CoS on the NF. As observed in Figs. 1f and k, the elemental mapping images of Co-MOF and Co-S confirm the presence of Co, Ni, and S elements.

**Figure 2:**
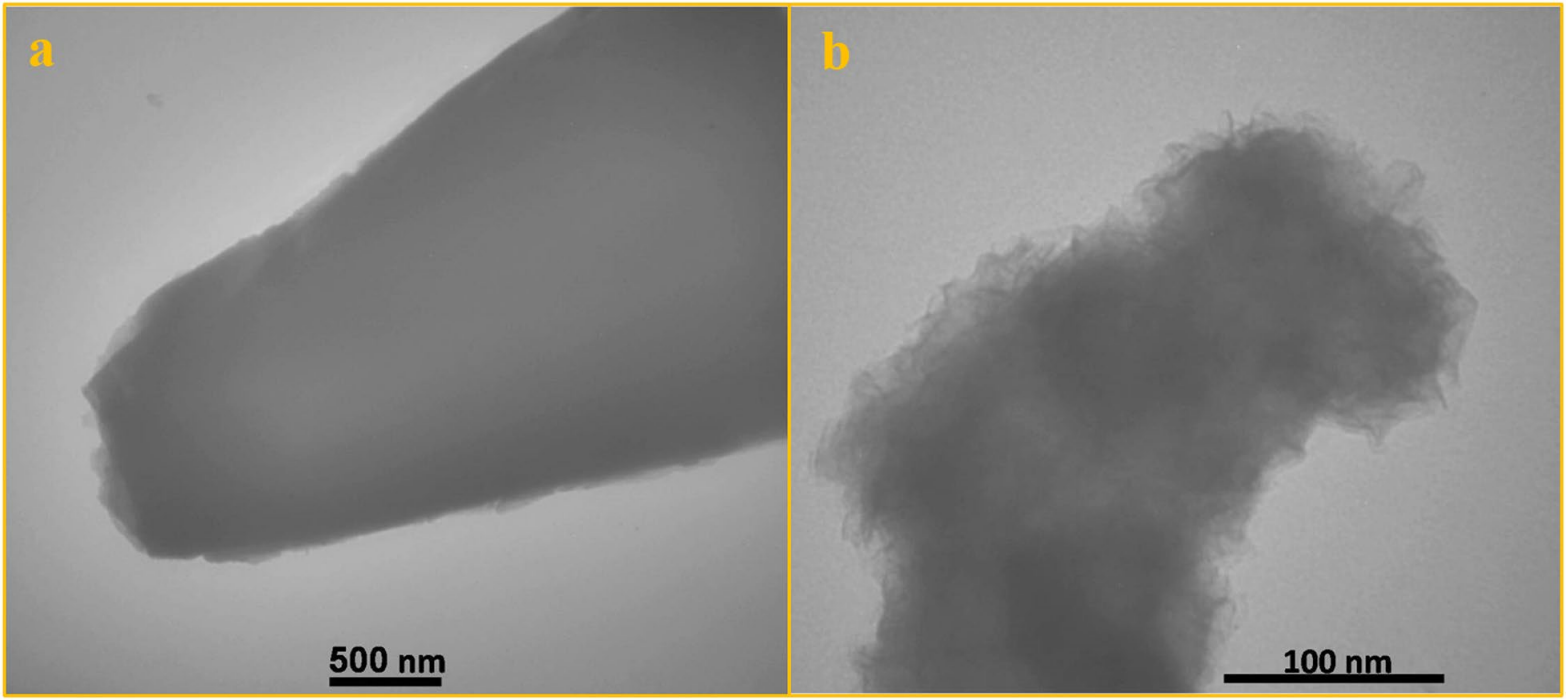
TEM images of (**a**) Co-MOF and (**b**) CoS.

TEM analyses were performed to further evaluate the structure of the prepared electrodes. In Fig. 2a, the Co-MOF nanosheets show a solid characteristic. Following sulfidation, the nanosheets maintain their structure with numerous nanoparticles distributed uniformly on the surface as observed in Fig. 2b. Furthermore, a mass of wrinkles can be observed, which is in good agreement with SEM results.

**Figure 3:**
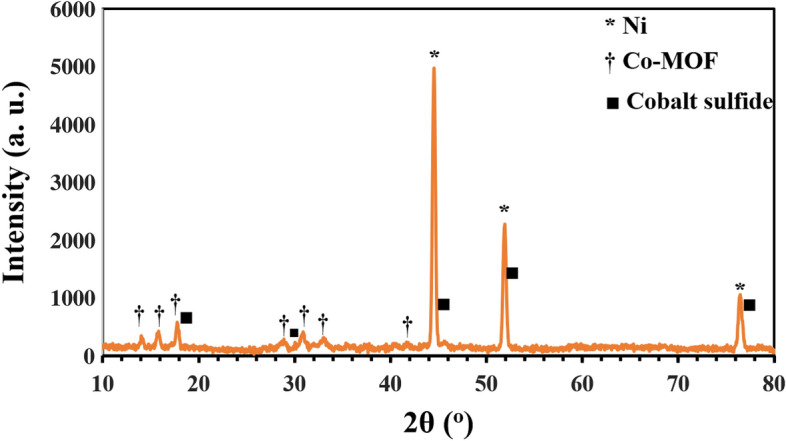
XRD patterns of Ni-foam, Co-MOF, and CoS electrodes.

The crystal phase of the synthesized electrode was determined via X-ray crystallography. Figure 3, shows the XRD patterns of NF, Co-MOF, and CoS samples. The three sharp and intense peaks observed at 2θ = 44.58, 51.82, and 76.35° are ascribed to the crystal planes of (111), (200) and (220), of cubic crystal phase of metallic NF substrate, respectively, according to reference JCPDS card number of 00–004-0850. The majority of remaining features of the XRD pattern well match with the reference JCPDS card number of 00–024-1644 for Co-MOF^36^. As Fig. 3 shows, the XRD peaks at 2θ = 17.85, 31.18, 44.68, 52.1, and 76.7° which are due to the cobalt sulfide crystal (JCPDS card number of 01-073-1442), overlap with Co-MOF and Ni foam peaks. The obtained results confirm the successful synthesis of CoS heterojunction on NF substrate.

**Figure 4:**
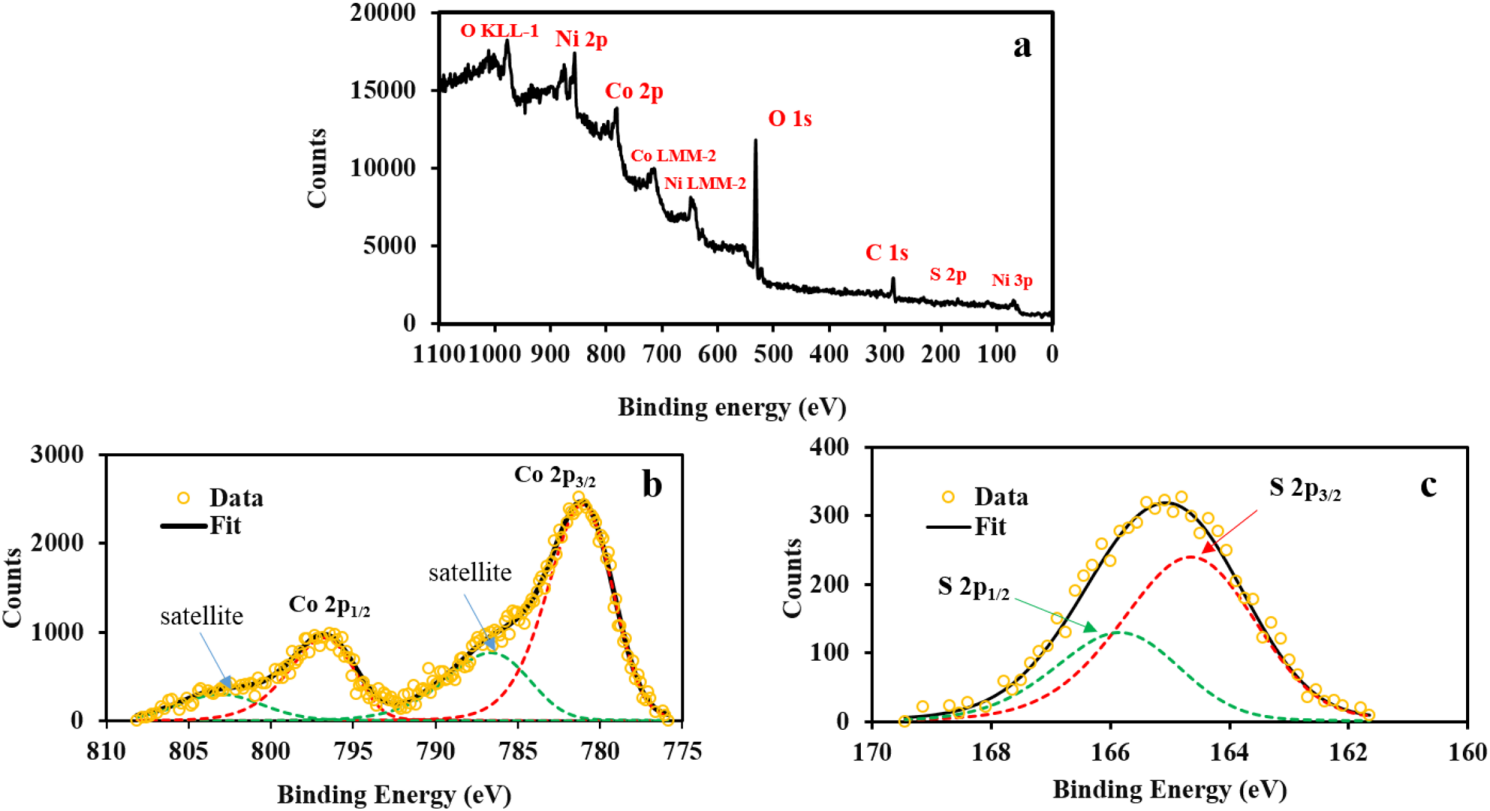
(**a**) Survey XPS spectra of NF, Co-MOF, and CoS electrodes, high resolution XPS spectra of (**b**) Co 2p and (**c**) S 2p core levels.

Figure 4a, shows the survey XPS spectra of NF, Co-MOF, and CoS electrode surfaces. The spectra confirm the presence of Ni, Co, O, C, and S elements, and no other impurity on the electrode surface. All these elements are also found in NF, Co-MOF, and CoS surface composition. The chemical state of Co and S more explored more via high resolution XPS spectra, which have been shown in Fig. 4. There are two peaks at 781.1 eV and 796.8 eV, with an energy difference of 15.7 eV in Co 2p spectrum (Fig. 4b). The corresponding satellite peaks are also observed at 786.7 and 803.1 eV, respectively. Such positions and spin-energy separation correspond to the Co 2p_3/2_ and Co 2p_1/2_ core levels and verify the Co^2+^ oxidation state in Co-MOF and CoS structures^37–39^. Figure 4c, shows the XPS spectrum of S 2p core level. Clearly, this peak has been fitted with two peaks located at 164.64 and 165.83 eV with a spin–orbit separation of 1.19 eV and relative intensities of 2:1, which are the characteristic features of S 2p_3/2_ and S 2p_1/2_ energy levels, respectively. The S 2p spectrum indicates that sulfur atoms are paired with the surrounding species, providing 2- formal valence state. Consequently, the Co atoms will have Co^2+^ oxidation state in CoS structure^40^.

**Figure 5:**
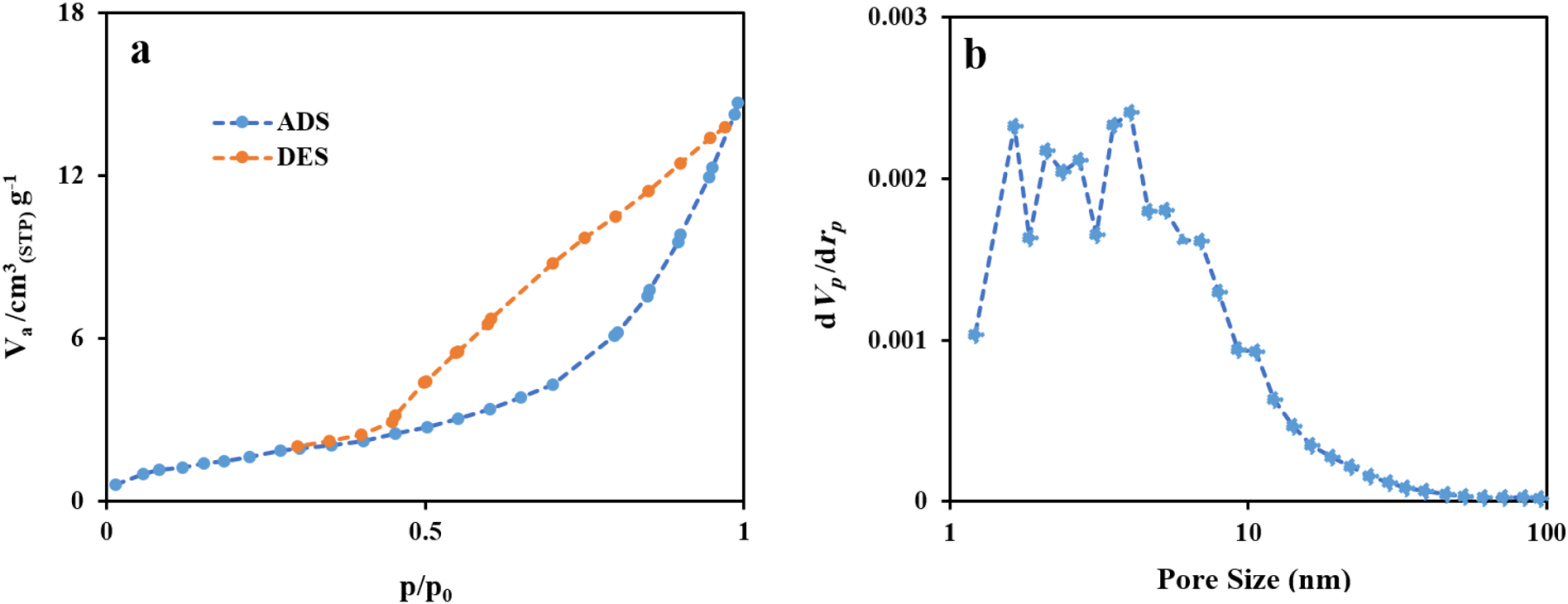
(**a**) The N_2_ adsorption–desorption isotherms and (**b**) BJH pore size distribution curve.

Next, to investigate the specific surface areas and porosity of the prepared electrode, N_2_ adsorption–desorption measurements were conducted. In Fig. 5, the obtained curves which correspond to the typical type IV isotherm with H3 hysteresis, indicate the presence of a mesoporous structure. The specific surface area of CoS was determined to be 142.93 m^2^ g^−1^ with an average pore diameter of ~ 4.03 nm. The pore structure of CoS is centered at the micro-mesoporous region. The microporous structures can provide a rich active site in the process of Faradic reaction and mesoporous structures provide an ion exchange channel. This is an important guarantee that the presence of microporous and mesoporous structures accelerates the ion transport rate at the electrode/electrolyte interface, as a result increasing the electrochemical performance of the electrode materials.

### Electrochemical evaluation of positive electrode for supercapacitor

**Figure 6:**
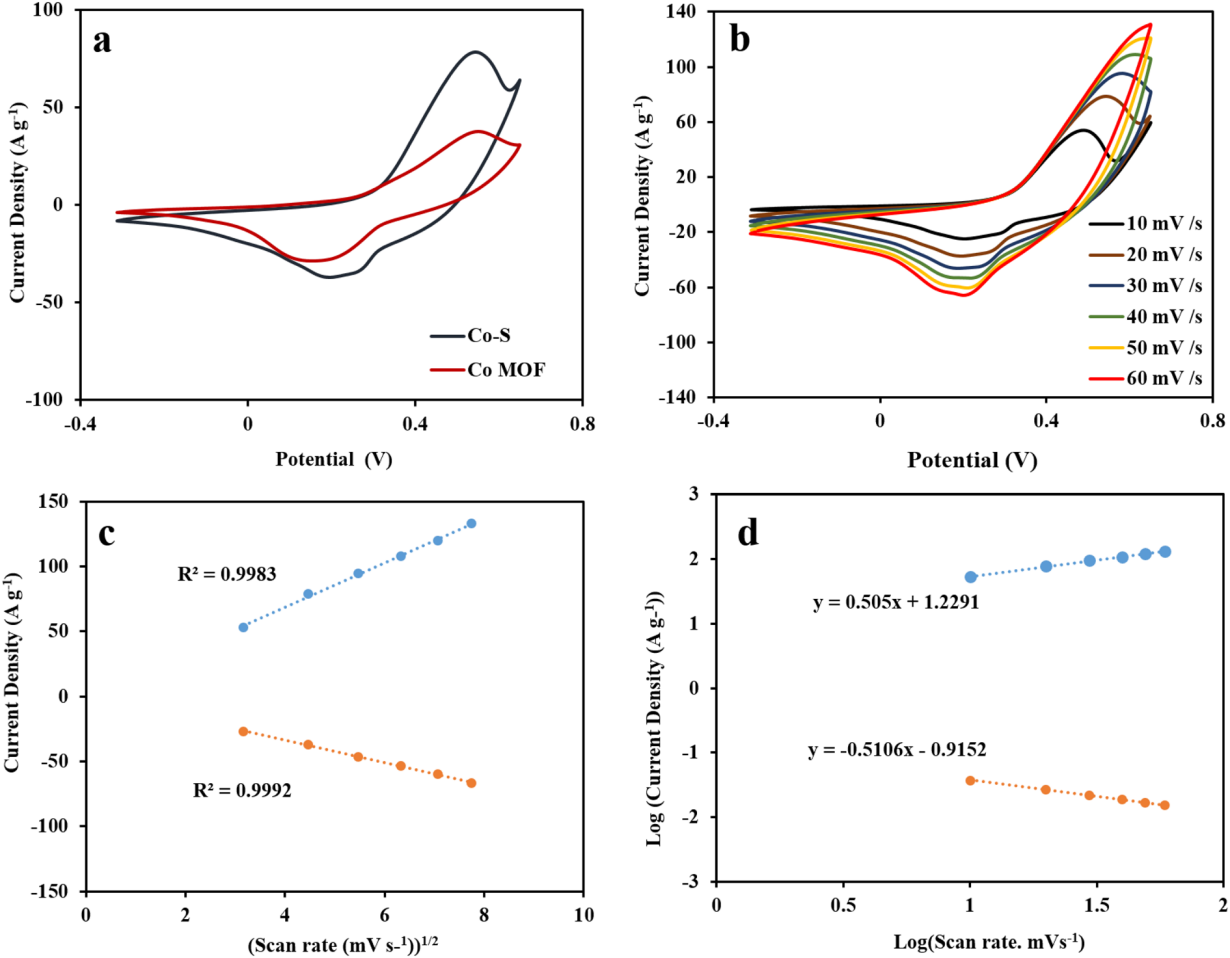
(**a**) CV curves of Co-MOF and CoS at 20 mV s^−1^, (**b**) CV curves of CoS at different scan rates, (**c**) linear curve of anodic and cathodic current density as a function of the square root of the scan rate, (**d**) Log (current density) vs. Log (scan rate) plot for CoS.

The electrochemical performances of Co-MOF and CoS modified electrodes were investigated using CV, GCD, and EIS techniques in a three-electrode configuration in a 3 M KOH electrolyte. The CVs of the Co-MOF and CoS at a constant scan rate of 20 mV s^−1^ are displayed in Fig. 6a. As shown in the CVs curves, one pair of quasi-reversible redox peaks is clearly observed for electrodes, indicating their faradic behavior. The CoS electrode exhibits a higher CV integrated area and peak current intensity compared to the Co-MOF electrode, indicating the higher capacitive performance of the former. The CV curves of the CoS electrode in 1 M, 3 M, and 6 M KOH electrolytes are shown in Fig. S1. The results verify the significant capacity of CoS electrode in 3 M KOH electrolyte. The CVs of Co-MOF and CoS were conducted at different scan rate ranging from 10 to 60 mV s^−1^ and the results are shown in Fig. S2 and Fig. 6b. It is obvious from the CV curves that the current density increases with increasing the scan rate, while the oxidation and reduction peaks are shifted to more positive and negative potentials, respectively, which can be attributed to the quasi-reversible nature of the redox reactions. The electrochemical performance of the CoS electrode can be adjusted by the sulfidation time. In this regard, different electrodes have been prepared under various reaction times of 5, 10, 20, and 40 min. According to the CV curves (Fig. S3), the electrode prepared at 10 min. Reaction time shows excellent specific capacity. For the CoS at 5 min, the ratio of CoS is low, indicating poor supercapacitor. By increasing the sulfidation time, the amount of CoS increases. However, the porous Co-MOF gradually disappears, leading to a less specific surface area, which results in a lower specific capacity.

Additionally, based on the Randles–Sevcik equation, a linear relationship is observed between the redox peak currents and the square root of the scan rate, confirming the diffusion-controlled behavior of the redox reactions at the interface of electrode/electrolyte (Fig. 6c). Figure 6d shows the Log(i)-Log(υ) plots for the anodic peak of the CoS electrode. The voltammetric response of the electrode was calculated according to equation Log i_p_ = a + b Log υ, in which i_p_ is the peak current (A), υ is the scan rate (V s^−1^), and (a) and (b) are constant parameters obtained from the mentioned plots (Fig. 6d). According to the b value, when b = 0.5, the current is diffusion controlled, whereas for b = 1, the current is adsorption (capacitive) controlled. Therefore, a value 0.502 for b (Fig. 6d), reveals the battery-type charge storage behavior of the CoS electrode.

**Figure 7:**
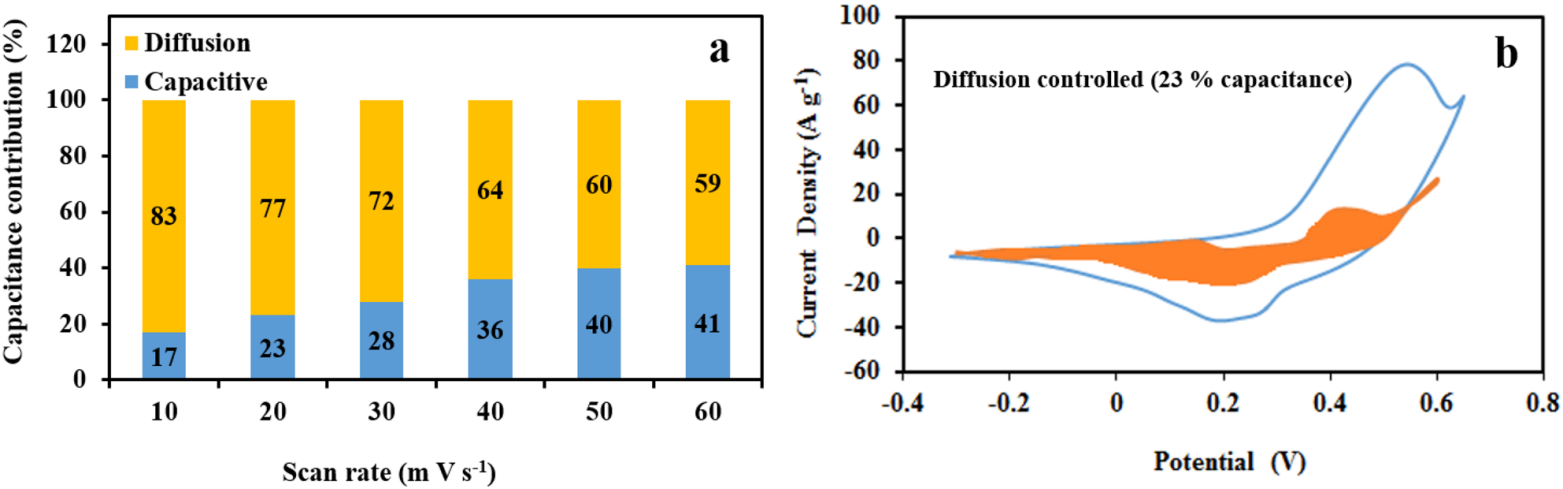
(**a**) Normalized contribution ratio of capacitive at different scan rates, (**b**) CV partition analysis showing capacitive contribution of CoS to total current at 20 m V s^−1^.

In addition, the capacitive and diffusion control contributions in the total current were calculated according to the equation I (V) = k_1_ υ + k_2_ υ^1/2^ (Doon method), where I is the current measured at a fixed potential (V), and k_1_ υ and k_2_ υ^1/2^ represent the capacitive and diffusion control contributions, respectively^41,42^. As shown in Fig. 7a and b, the calculated capacitive contributions are 17%, 23%, 28%, 36%, 40%, and 41% at the scan rates of 10, 20, 30, 40, 50, and 60 mVs^−1^, respectively. As the scan rate is increased, the surface dependent capacitive behavior becomes more dominant because of the time limitation imposed by higher scan rates on the ionic transport of electrolyte ions within the surface active materials.

**Figure 8:**
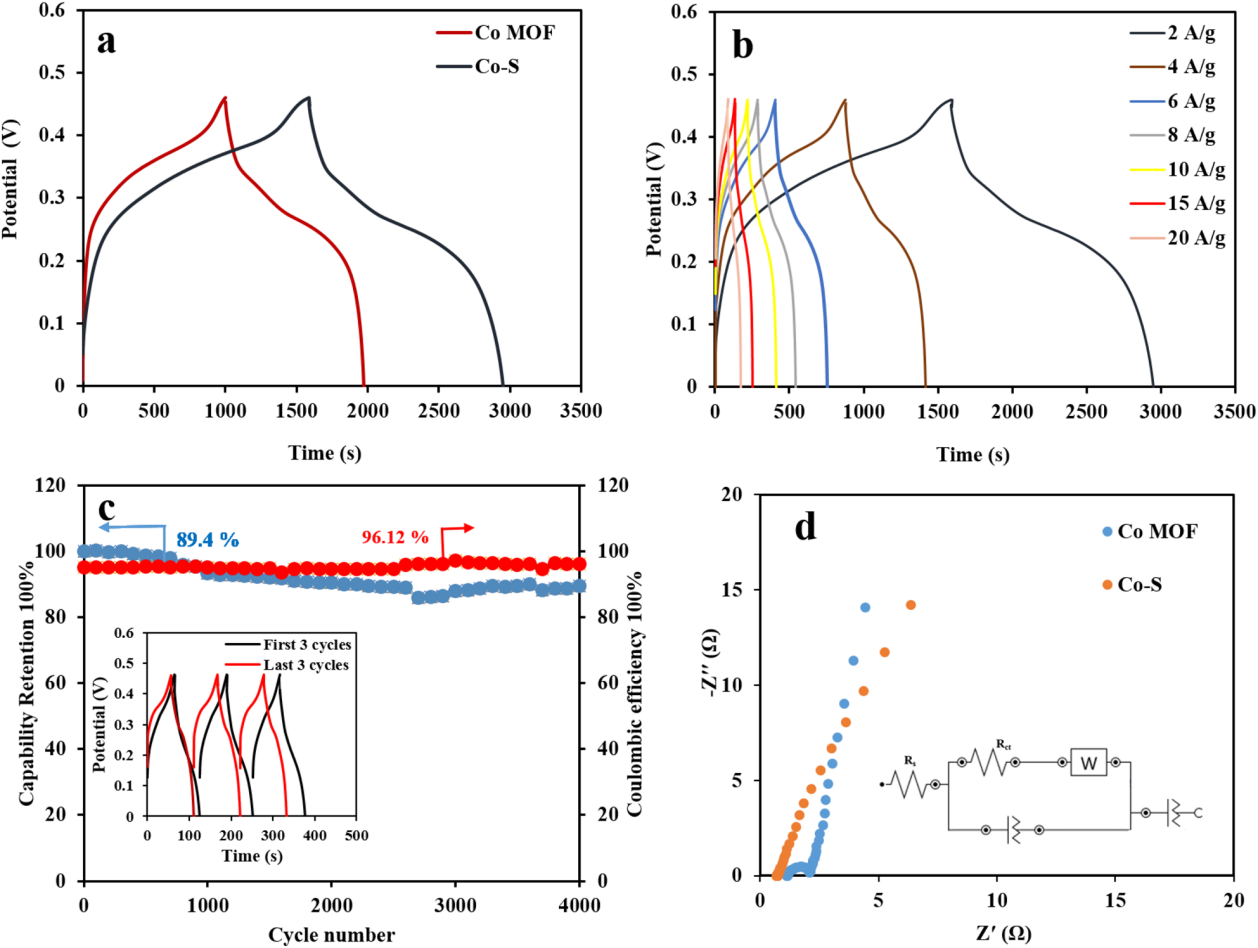
(**a**) GCD curves of Co-MOF and CoS electrodes at a current density of 2 A g^−1^, (**b**) GCD curves of Co-S electrode at different current densities, (**c**) capacity cycling performance and coulombic efficiency of CoS electrode at a current density of 20 A g^−1^, (inset shows the first 3 and last 3 cycles), (**d**) Nyquist plots of CoS and Co-MOF.

The GCD results of the Co-MOF and CoS electrodes at a current density of 2 A g^−1^ are shown in Fig. 8a. In accordance with the CV results, CoS/NF electrode indicates a battery-type nature and longer discharge time than the Co-MOF electrode. The GCD curves of CoS electrode in 1 M, 3 M, and 6 M KOH electrolytes and various sulfidation times are shown in Figs. S4 and S5. The GCD curves of Co-MOF and CoS/NF electrodes at various current densities from in the range of 2–20 A g^−1^ exhibit excellent symmetry with an IR-drop (Fig. S6 and Fig. 8b). The large IR-drop can be related to the poor electrical conductivity due to the presence of Co-MOF. As shown in Fig. 8b at a high current density of 20 A g^−1^, CoS/NF electrode can retain 64.1 ℅ of its initial capacity, indicating its good rate capability. The capacity values for CoS electrode are 377.5 mA h g^−1^ (1359 C g^−1^), 300.5 mA h g^−1^ (1090 C g^−1^), 292.25 mA h g^−1^ (1052.1 C g^−1^), 282.4 mA h g^−1^ (1016.8 C g^−1^), 275.1 mA h g^−1^ (990.5 C g^−1^), 252.7 mA h g^−1^ (909.75 C g^−1^), and 242 mA h g^−1^ (870 C g^−1^), at 2, 4, 6, 8, 10, 15, and 20 A g^−1^, respectively. The cycling stability of CoS electrode was evaluated by GCD method at a high current density of 20 A g^−1^ in the potential range of 0–0.45 V during 4000 cycles. The results show that 89.4% of the initial capacity is retained after 4000 cycles, with a high coulombic efficiency (η) of indicating good reversibility and impressive cycling capability (Fig. 8c). The insert exhibits the first and last three cycles of the CoS modified electrode.

EIS measurements were conducted to evaluate the charge transfer kinetics and diffusion properties of the prepared electrode. Figure 8d shows the Nyquist plots of Co-MOF and CoS electrodes at the frequency range of 0.1 Hz to 100 kHz at open circuit potentials for better insight of the electrochemical performance of the as-fabricated electrodes. Each curve consists of a semicircle in high frequency regions (R_ct_) and a straight line in the low frequencies (R_s_). The semicircle at high frequency region can be attributed to the charge transfer resistance and a straight line at low frequency region is ascribed to the diffusion resistance at the interface between the electrode and electrolyte^43,44^. The R_s_ and R_ct_ values were 0.7 and 0.04 Ω for CoS and 1.1 and 1 Ω for Co-MOF electrode, respectively. As shown, the semicircle is smaller for CoS electrode. In addition, the slope of the straight line at low frequencies for CoS electrode is steeper than that of Co-MOF/NF, indicating that the former electrode has a smaller interfacial charge transfer and diffusion resistances. These results can be related to the higher specific capacity of CoS and facilitated diffusion of electrolyte ions, compared to the other electrode. The Bode phase angle plots of CoS and Co MOF electrodes are shown in Fig. S7. The Nyquist plots of CoS before and after 4000 cycles are displayed in Fig. S8. A longer Warburg line after 4000 cycles implies the batter electrolyte diffusion into the electrode material compared with the initial cycle.

### Electrochemical performance of CoS//AC device

After fabricating and characterizations in3-electrode systems, an ASC device consisting of CoS//AC was constructed. This asymmetric device consists of CoS and AC as the positive and negative electrodes, respectively, and 3 M KOH solution as the electrolyte. Using CV and GCD methods, the energy storage of this ASC device was investigated. AC/NF indicates a typical electric double-layer capacitance property in the −1 to 0 V range in various scan rates and current densities (Fig. S9). The specific capacitance value for AC electrode are 297, 232, 211, 181, and 136 Fg^−1^ at 2, 4, 6, 8, and 10 A g^−1^, respectively.

**Figure 9:**
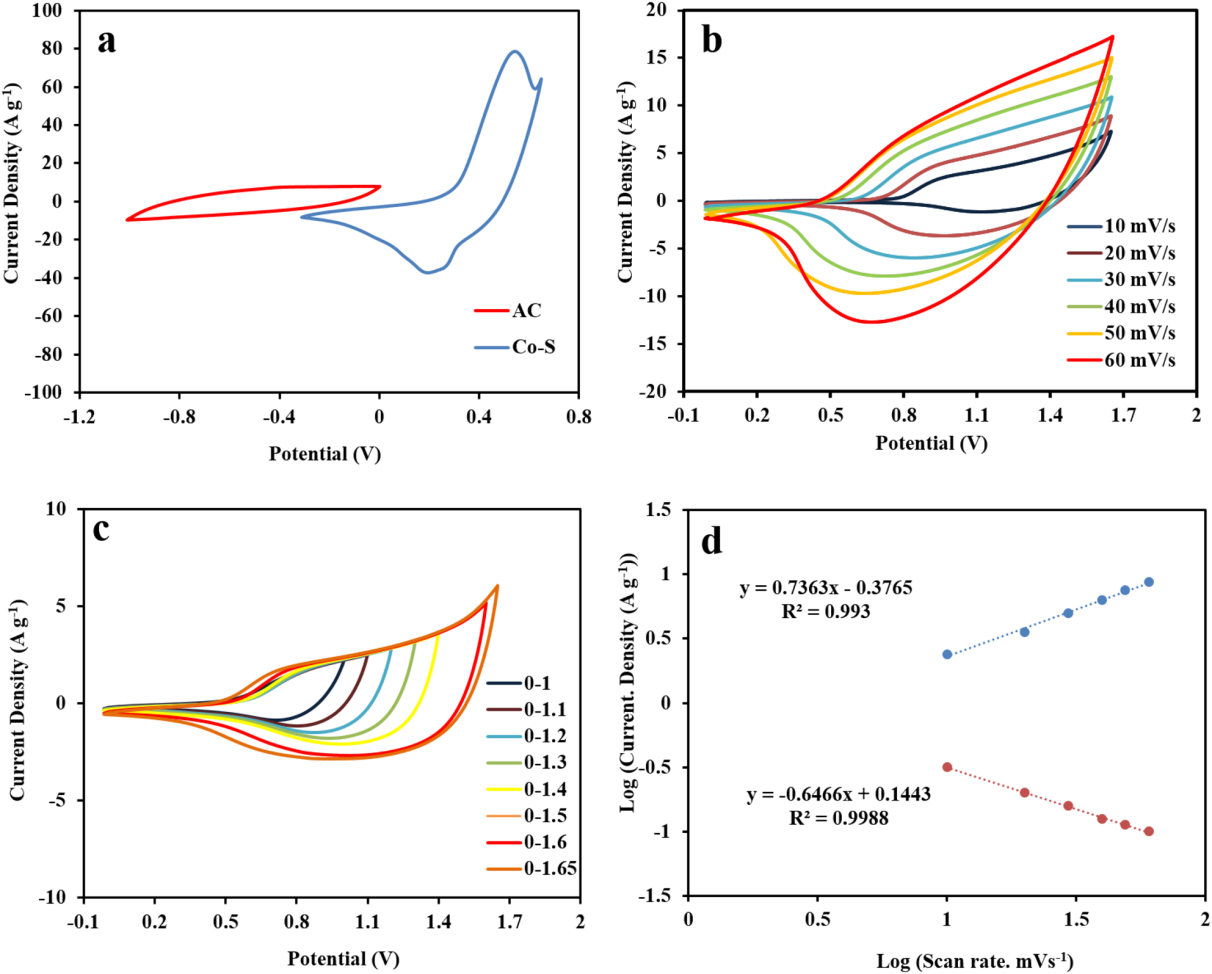
(**a**) CV plots of CoS and AC electrodes at a current density of 2 A g^−1^ in the separate three electrode systems, (**b**) CV curves at different scan rates, (**c**) CV curves of CoS//AC asymmetric supercapacitor at different potential windows at a scan rate of 20 mV s^−1^, (**d**) the Log (Current Density) vs. Log (Scan Rate) plot for CoS//AC asymmetric supercapacitor.

To show the working potential range, the CVs of AC and CoS electrodes, after mass balance were recorded in a 3-electrode system at a scan rate of 20 mV s^−1^ (Fig. 9a). The potential ranges were -1 to 0 and 0.3 to 0.65 V for AC and CoS, respectively. The CVs curves of the asymmetric CoS//AC device at different scan rates in the range of 10–70 mV s^−1^ over a potential range of 0–1.65 V are shown in Fig. 9b. The CV curves of CoS//AC device were recorded in the potential range 0–1 V to 0–1.65 V at a scan rate 20 mV s^−1^ in (Fig. 9c). It can be inferred from the CV results that there are both Faradaic and capacitive contributions toward the load storage system. The fabricated asymmetric supercapacitor device displays a typical capacitance behavior with quasi-rectangular CV curves without any clear redox peaks. The b-value for the anodic and cathodic peaks is 0.73 and 0.64, respectively (Fig. 9d). This shows an intermediate behavior between psudocapacitive and diffusive faradic processes, in which a clear boundary is not easy to define^45^.

**Figure 10:**
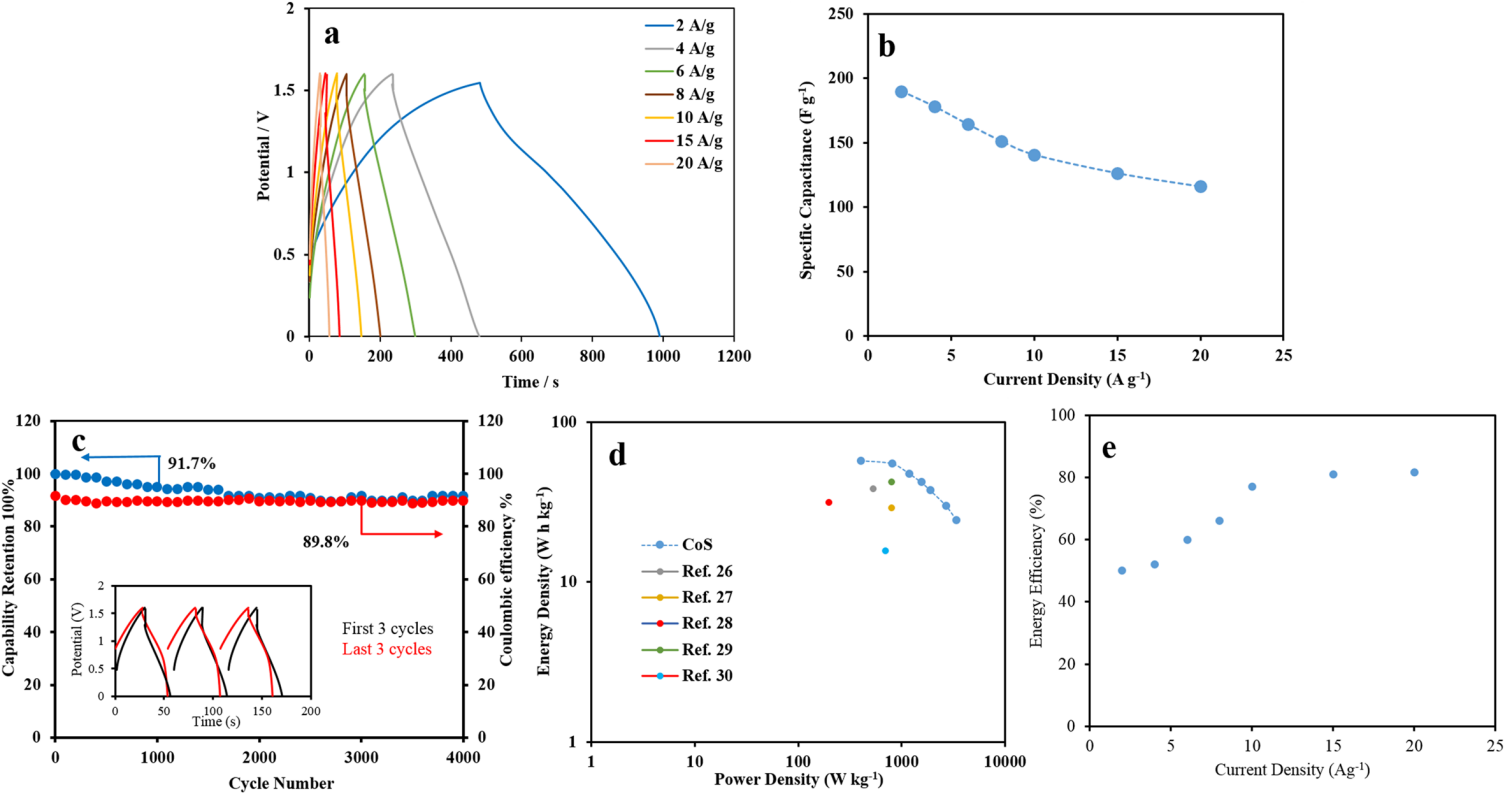
(**a**) GCD curves of the asymmetric device at different current densities, (**b**) dependence of the gravimetric capacitance of the fabricated ASC on the applied current density calculated from the GCD curves, (**c**) cycling performance and coulombic efficiency of the device at current density of 20 A g^−1^ over 4000 cycles (inset shows the first and last 3 cycles), (**d**) Ragone plot of device, and (**e**) energy efficiency of CoS//AC asymmetric supercapacitor.

The capacitive behavior of the prepared ASCs was investigated by the GCD method. Figure 10a shows the GCD results of the asymmetric supercapacitor devices within a potential range of 0 to + 1.6 V at various current densities from 2 to 20 A g^−1^. The results indicate that the asymmetric CoS//AC device has both capacitive and Faradic contributions. A noteworthy specific capacitance of 174.3 F g^−1^ at a current density 2 A g^−1^ is observed for the asymmetric CoS device, due to both the porous and spongy structure of AC and the presence of cavities and large CoS surface. The specific capacitance of ASC based on the total mass of active materials and discharge time, is shown in Fig. 10b. The capability of ASC to maintain a capacitance of 108 F g^−1^ at a current density of 20 A g^−1^ shows its good performance at high currents. Figure 10c shows the cycling stability of ASC at the current density of 20 A g^−1^ and 91.7% retention of capacitance after 4000 cycles. The insert indicates the first and last three cycles of the prepared electrode. Furthermore, the coulombic efficiency of The CoS//AC electrode was calculated from the charge and discharge times (Fig. 10c). The Nyquist plots of CoS//AC before and after 4000 cycles are displayed in Fig. S10. The comparison of the results obtained with those of other reports (Table 1) further shows the excellent specific capacitance and cyclic stability of this battery-type CoS electrode in energy storage. As shown in Fig. 10d the Ragone plots indicate the high energy density of 57.4 W h kg^−1^ at the power density of 405.2 W kg^−1^. For more realistic characterization, the energy efficiencies are reported in Fig. 10e. These values are comparable or superior to those of cobalt-based supercapacitors previously reported.

## Conclusion

In summary, we have successfully developed a simple two-step strategy to synthesize the desirable CoS on NF from Co-MOF. The Co-MOF nanosheets are first generated by the solvothermal method and partially transfer to CoS by the water bath process 80 °C. The CoS electrode has been found to deliver a remarkable electrochemical performance when operated in combination with an electrolyte (3 M KOH). The optimized electrode/electrolyte system (CoS/3 M KOH) has yielded a highest specific capacity 377.5 mA h g^−1^ (1359 C g^−1^) at the current density of 2 A g^−1^. Also the specific surface area and electrical conductivity of the resultant CoS are influenced by the sulfidation time. These items result in the diverse electrochemical response of CoS samples as electrode materials for supercapacitor. The CoS obtained at time of 10 min give rise to ideal capacitor behaviors d to their good electrical conductivity. The particular structure exhibited a prominent cycling stability of 89.4% after 4000 cycles. A hybrid supercapacitor is also successfully assembled using CoS/NF as the positive electrode and activated carbon as negative electrode, it exhibits a high energy density 57.4 W h kg^−1^ at a power density of 405.2 W kg^−1^. In addition, the present materials can be as promising candidate materials available for the potential multifunctional applications of energy storage systems.

### Supplementary Information

Supplementary Figures.

## Data Availability

The datasets used and/or analyzed during the current study are available from the corresponding author on reasonable request.
