# Supplementary material for: Cobalt sulfide flower-like derived from metal organic frameworks on nickel foam as an electrode for fabrication of asymmetric supercapacitors
Source: Sci Rep. 2024 Mar 13;14:6045. doi: 10.1038/s41598-024-56689-9 (PMC10933289; doi:10.1038/s41598-024-56689-9)
Supplement: Supplementary file 1 — Supplementary Figures. [file 41598_2024_56689_MOESM1_ESM.docx]

**Electronic Supplementary Material**

Cobalt sulfide flower-like derived from metal organic frameworks on nickel foam as an electrode for fabrication of asymmetric supercapacitors

Farzaneh Nasiri^a^, Lida Fotouhi^a, b*^, Saeed Shahrokhian^c^, Mohammad Zirak^d^

*^a^Department of Analytical Chemistry, Faculty of Chemistry, Alzahra University, Tehran, Iran*

*^b^Analytical and Bioanalytical Research Centre (ABRC), Alzahra University, Tehran, Iran*

*^c^Department of Chemistry, Sharif University of Technology, Tehran 11155–9516, Iran*

*^d^Department of Physics, Hakim Sabzevari University, P. O. Box 961797647, Sabzevar, Iran*

***
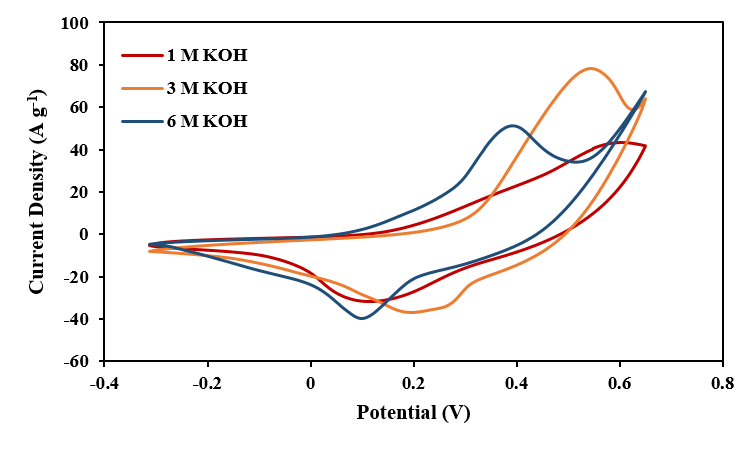
***

**Fig. S1.** The CV curves of the CoS electrode in 1M, 3M and 6M KOH electrolytes.


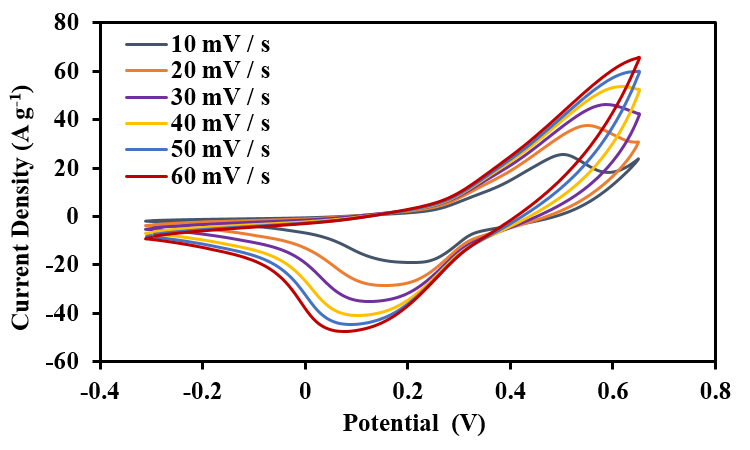


**Fig. S2.** The CVs of Co-MOF at the different scan rates.


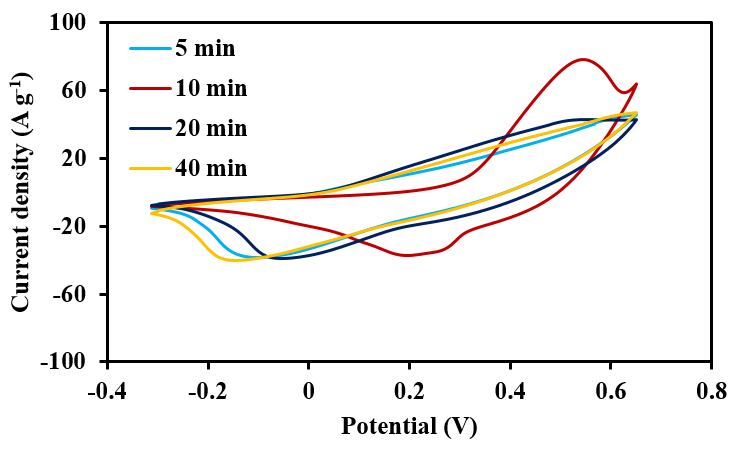


**Fig. S3.** The CVs of CoS electrode in various reaction time.


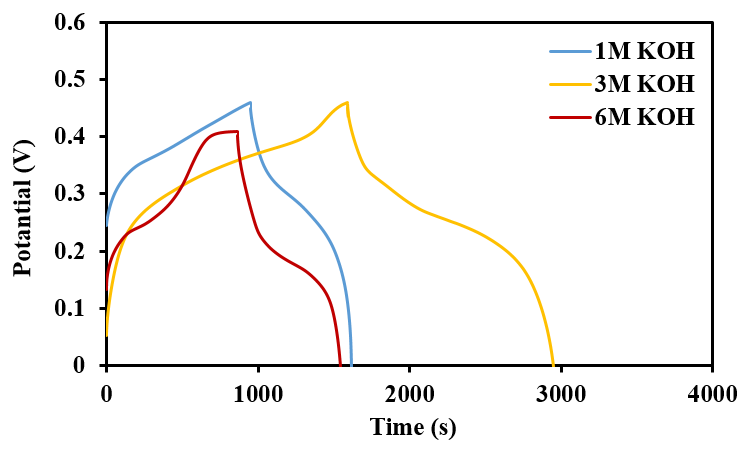


**Fig. S4.** The GCD curves of Co-S electrode in 1M, 3M and 6M KOH electrolytes


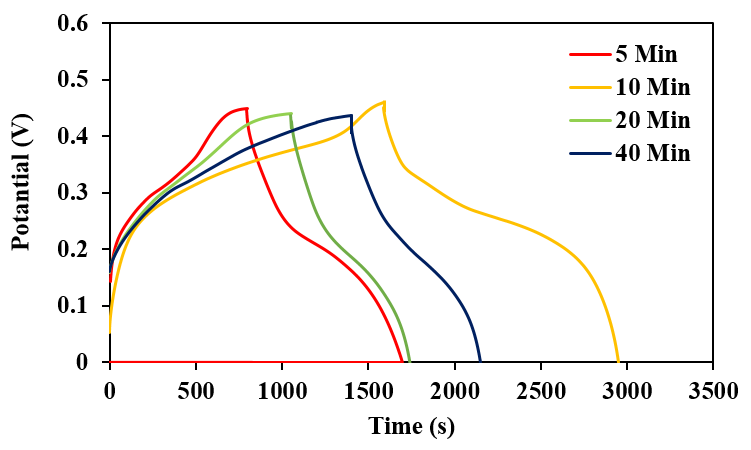


**Fig. S5.** The GCD curves of Co-S with various sulfidation times


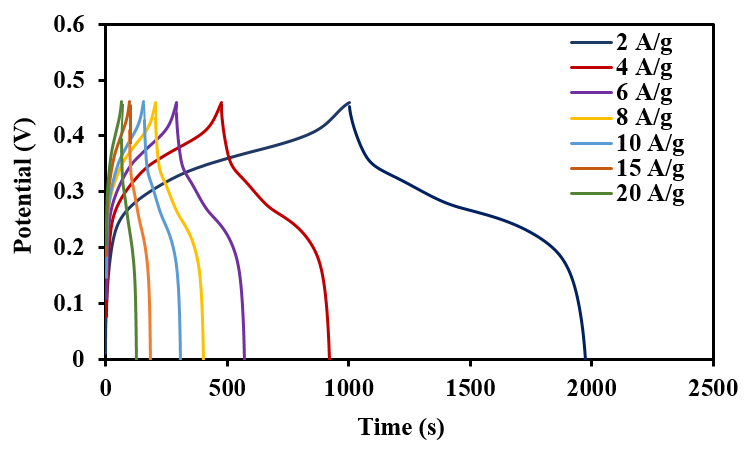


**Fig. S6.** The GCD curves of Co-MOF electrode at various current densities.

**Fig. S7. Bode phase angle plot of CoS and Co MOF electrodes**

**Fig. S8.** The Nyquist plots of CoS before and after 4000 cycles.


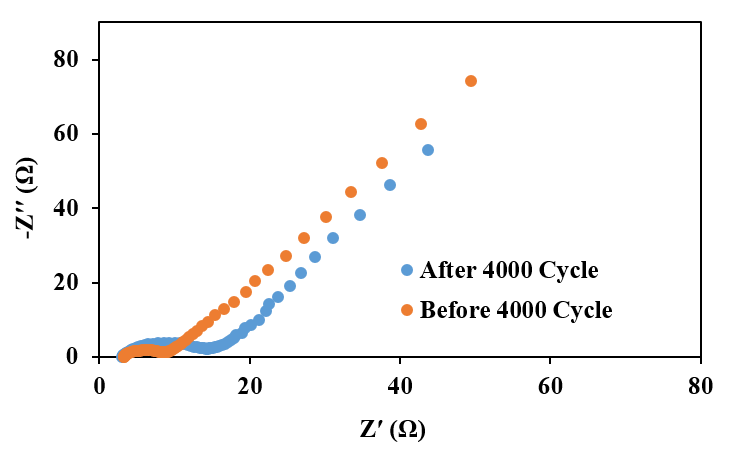


**Fig. S9.** The Nyquist plots of CoS**//AC** before and after 4000 cycles.
